# Individual and community level factors of malaria among under-five children in Kenya: Based on the Kenya Demographic and Health Survey

**DOI:** 10.1371/journal.pone.0335346

**Published:** 2025-10-27

**Authors:** Gebeyehu Lakew, Abiyu Abadi Tareke, Zeamanuel Anteneh Yigzaw, Demis Getachew, Eyob Getachew, Amlaku Nigusie Yirsaw

**Affiliations:** 1 Department of Health Promotion and Health Behavior, School of Public Health, College of Medicine and Health Sciences, University of Gondar, Gondar, Ethiopia; 2 Zonal-Level COVAX and Routine Immunization Technical Assistance (TA) at West Gondar Zonal Health Department, Gendawuha, Ethiopia; 3 Health Promotion and Behavioral Science Department, School of Public Health, College of Medicine and Health Science, Bahir Dar University, Bahir Dar, Ethiopia; 4 Department of Pharmacology, School of Pharmacy, College of Medicine and Health Sciences, University of Gondar, Gondar, Ethiopia; Freelance Consultant, Myanmar, MYANMAR

## Abstract

**Background:**

Malaria remains a significant global public health challenge. In Kenya, it is a leading cause of illness and death among children under five in malaria-endemic areas. Understanding its prevalence and the factors influencing it is essential for informed public health decisions and targeted interventions.

**Objective:**

Assess prevalence and associated factors of malaria in under-five children using the 2022 Kenya Demographic and Health Survey data.

**Method:**

A total of 3146 children were included in this study. A multilevel logistic regression model was fitted to assess factors associated with malaria, with significance reported at p-value < 0.05 and a 95% confidence interval.

**Results:**

The prevalence of malaria among under-five children in Kenya was 22.72% (95% CI: 21.29, 24.22). Maternal age between 25–34 (AOR:0.75,95% CI:0.57,0.99) years, richest wealth of family (AOR 0.48, 95% CI 0.26–0.89), treated mosquito bed net (AOR = 0.39; 95% CI: 0.17, 0.89), no bed net (AOR = 0.49; 95% CI: 0.31, 0.77), mothers who are breast feeding (AOR = 0.66; 95% CI: 0.52, 0.85) and community level poverty(AOR = 1.82; 95% CI: 1.19, 2.77) were the associated factors of malaria among underfive children in Kenya.

**Conclusion:**

Malaria prevalence among under-five children in Kenya was 22.72%, with risk factors including poverty and lack of bed net use, while maternal age (25–34 years), higher wealth, treated bed nets, and breastfeeding were protective. To reduce malaria, promoting the use of insecticide-treated bed nets, supporting low-income families, and strengthening maternal health education are essential. Additionally, poverty alleviation programs and enhanced malaria surveillance can help mitigate risks. Strengthening community-based interventions and improving healthcare access are crucial for long-term malaria control.

## Introduction

Malaria is a persistent global health challenge and a significant public health concern in many countries, including Kenya [1]. It remains endemic in over 100 countries, placing nearly half of the global population at risk, with approximately one million deaths occurring annually due to the disease [2]. This life-threatening illness is transmitted indirectly among humans through the bite of female Anopheles mosquitoes, which serve as vectors for one of the five Plasmodium parasite species [3].

In Africa, malaria transmission tends to be higher in rural areas than in urban settings, likely due to increased vector density, lower-quality housing, and inadequate drainage systems [3]. In Kenya, malaria continues to pose a major public health challenge, contributing to high morbidity and mortality, with over 70% of the population at risk of infection [4]. However, the disease burden is not uniform across the country, with regions around Lake Victoria and the Coastal areas experiencing the highest risk. Children under the age of five remain the most vulnerable to infection [5,6]. Previous studies conducted in Kenya have reported varying malaria positivity rates, including hospitalized cases, generally ranging from 4.8% to 70% among children under five years [1,7].

Previous studies on childhood malaria in Kenya have primarily been limited to research conducted in hospitals or clinics (institutional level) or have been focused on specific rural regions, tackling only personal risk factors for individual children (individual level). This new research aims to offer a more thorough understanding by investigating both individual and community-level influences that contribute to childhood malaria throughout Kenya. This current study tackles this limitation by utilizing a more sophisticated method, multivariable multilevel logistic analysis, which takes into account data clustering. This strategy enables more precise and dependable outcomes as it offers a more thorough perspective on the data.

This study aims to fill that gap by investigating the multilevel determinants of malaria among children under five using recent nationally representative data. By examining both individual and community contexts, the study seeks to inform more targeted and effective public health interventions for malaria prevention and control in Kenya.

## Methods

### Study area, data source, and study period

The Kenya Demographic and Health Survey (KDHS) 2022 took place from February 17 to July 13, 2022. This survey offers extensive information on demographic, health, and nutrition metrics throughout Kenya, acting as an essential tool for policy development, program execution, and research in the nation’s health sector [8].

In Kenya, the administrative structure is organized hierarchically, with several levels influencing governance and service delivery. The highest level is the national government, which oversees national policies and strategic goals. Below this, Kenya is divided into 47 counties, each functioning as a first-level administrative unit. Each county is governed by an elected governor and county assembly, which manage local resources and services. These counties serve as the second-level administrative units in the multilevel analysis. Each county is further subdivided into sub-counties, which serve as third-level administrative units, with a total of 290 sub-counties across the country. These sub-counties are divided into divisions, which are responsible for the local implementation of policies. Beneath the subcounty level, there are 1,450 wards, which represent the fourth level of administration. Each ward is headed by an elected representative and plays a key role in local governance and service delivery. The smallest administrative units in Kenya are the villages, which are grassroots units that serve as the community level for decision-making and interaction. There are approximately 30,000 villages in Kenya, each representing a local community with varying degrees of governance influence. By analyzing this hierarchical structure, the study will assess the impact of governance and resource allocation at the national, county, sub-county, ward, and village levels, allowing for a nuanced understanding of how administrative levels contribute to development outcomes [8].

### Sampling technique and study population

The KDHS utilized a carefully organized sampling method, relying on the enumeration areas defined during the 2022 Population and Housing Census of Kenya as the primary sampling framework. This strategy guarantees the representativeness of the survey results by utilizing the detailed and current data collected during the nationwide census [9].

The comprehensive sampling design of the 2022 Kenya Demographic and Health Survey (KDHS) ensures a nationally representative dataset covering both urban and rural areas across all 47 counties. The study selected 1,692 clusters (enumeration areas), including 666 urban and 1,026 rural clusters, and systematically sampled 25 households per cluster, resulting in a total sample of 42,300 households. Among these, 37,911 households were successfully interviewed, ensuring a high response rate. To assess malaria prevalence among under-five children, mothers or caregivers were asked whether their child had experienced fever in the two weeks preceding the survey. If the child had a fever, further questions were asked about whether they received treatment and whether a diagnostic test for malaria was performed. This approach provides crucial insights into malaria burden and healthcare-seeking behavior, guiding targeted interventions such as improved access to malaria treatment, increased use of insecticide-treated nets, and community-based awareness programs [9].

In this study, the total sample includes 3,146 children. It’s important to recognize that this sample is weighted; this process adjusts the data to reflect the intricate survey design and ensure it is representative of the whole population. This thoughtful approach to sampling and the subsequent weighting of the data improves the validity and reliability of the results, offering essential insights into the health and demographic trends of children under five in Kenya as of 2022 [9].

### Study variables

#### Dependent variable.

The dependent variable in this study was malaria infection status among children under five years of age, as recorded in the Kenya DHS dataset. Malaria was tested using Rapid Diagnostic Tests (RDTs) conducted in the field during the survey. Capillary blood was collected via finger prick, and RDTs were used to detect *Plasmodium falciparum*-specific antigens [8].

The outcome was recorded as a binary variable:

1 = Positive for malaria (RDT result positive)

0 = Negative for malaria (RDT result negative)

Only children with valid RDT test results were included in the analysis. Children without a test result or with missing values were excluded.

#### Independent variables.

The independent variables were selected based on a thorough review of existing literature, theoretical relevance, and their availability in the Kenya DHS dataset.

At the individual level, factors examined for their link to malaria in Kenya encompassed the child’s age, child’s gender, maternal age, maternal education level, socioeconomic status, type of bed net, utilization of a bed net during the child’s sleep, maternal employment status, the number of children under five, breastfeeding status, delivery location, and exposure to media. In addition, factors at the community level, including the overall education and poverty levels of the community, were also considered.

***Operational definition*:** **Media exposure**: This research grouped the three media types, newspapers, radio, and television, into a unified category termed “exposed to media” to capture a wider array of participants. Individuals were classified in this category if they indicated usage of any of the media types. This strategy helps to mitigate the risk of excluding participants that could occur if each media type were examined independently. For example, certain questions might only be relevant to specific demographics, such as newspapers for educated women or television for individuals with access. By consolidating the data, the research guarantees a more inclusive and thorough analysis [10].

**Community-level maternal poverty**: Community-level poverty was determined by the proportion of women in the poor and poorest quintile. It was coded as “0” for low (communities in which < 50% women had poor and poorest wealth quintiles), “1” for high (communities in which ≥ 50% women had poorest and poorer wealth quintiles) poverty communities [11].

**Community-level maternal educational status:** The research additionally investigated the educational level in various communities by analyzing the share of mothers or caregivers who had completed at least a primary education. Communities where a larger percentage of mothers or caregivers achieved primary education or above were labeled as having a “high community level of educational status.” In contrast, communities where the majority of mothers or caregivers possessed less than a primary education were identified as having a “low community level of educational status.” This approach effectively assesses the general educational attainment within a specific community [11].

### Data management and analysis

This research highlighted the main traits of the data through the use of medians, tables, and percentages. We refined the data in Excel to guarantee its precision. To tackle any disparities in the representation of various groups within the sample, we utilized weighted frequencies. In essence, we modified the data to mirror the proportional representation of each group within the broader population.

### Mixed model

This research employed a unique technique known as multilevel logistic regression to identify factors associated with malaria in Kenya. This approach is especially effective for examining data that has hierarchical structures, such as the information from the Demographic and Health Survey (DHS).

The DHS data includes information on both specific children and the communities where they reside. For instance, they possess details regarding each mother’s educational background and the economic status of the household. The researchers anticipate that mothers who have attained a higher level of education and income will have children who are less likely to contract malaria [12].

Nevertheless, the analysis extends beyond merely looking at personal attributes. It additionally examines how factors at the community level could impact these individual characteristics. For example, a mother’s level of education and her income may be influenced by her residential area (enumeration area), the general media exposure within her community, the community’s level of poverty, and even the geographical region of the country. By incorporating these community-related factors, the research can offer a more thorough understanding of the elements contributing to childhood malaria [13].

The information utilized in this research originates from the Demographic and Health Survey (DHS), which gathers data at both the individual and community levels. This results in a hierarchical structure, whereby children within the same community (enumeration area) are expected to exhibit greater similarity than those from different communities. This shared similarity in traits (such as education or wealth) breaches the assumption of independence expected in conventional regression models [13].

Multilevel logistic regression analysis is tailored to tackle this problem. It considers the hierarchical arrangement of the data, enabling us to investigate the elements linked to malaria while recognizing that children from the same community may share more similarities. This approach gives a more precise understanding of how both individual and community-level factors influence childhood malaria [12].

This research employed a two-level binary logistic regression model to investigate the factors associated with malaria in Kenya. This model accounts for both individual and community-level variables. The researchers analyzed four distinct models. The initial model, referred to as the “null model,” did not include any explanatory variables. Its purpose was to measure the extent of variation in malaria prevalence among different communities. This model partitions the total variance into within-cluster and between-cluster components, allowing us to examine whether there is significant variability in malaria prevalence at the community level.

The presence of clustering was assessed using the Intra-Class Correlation Coefficient (ICC), which quantifies the proportion of total variance attributable to differences between clusters. A significant ICC value justified the use of multilevel modeling, indicating that malaria risk varied significantly across communities and that individual-level analysis alone would be insufficient. To determine the best-fitting model, they examined various criteria: the Likelihood Ratio test (LLR), deviance, Akaike information criterion (AIC), and deviance information criterion (DIC). The model with the lowest score in one of these four criteria was selected as the most accurate depiction of the data. In other words, this model most effectively elucidates the factors linked to childhood malaria [13].

Ultimately, the median odds ratio (MOR) offered a new viewpoint. It indicated the extent to which the probability of contracting malaria could vary between communities at the highest and lowest risk levels. The values of ICCs and MORs were derived from intercept-only models (null model) to assess the presence of clustering and variability of malaria cases across different areas. Lastly, variables with a p-value less than 0.25 were selected for multivariate analysis. An adjusted odds ratio with a 95% confidence interval (CI) and a p-value below 0.05 was used to determine statistical significance.

### Weighting

Since the sample for this study is a two-stage stratified cluster sample, level weights were calculated separately, based on sampling probabilities for each sampling stage and cluster. In this study, level weights were estimated using a framework for approximating level weights in Malaria Indicator Surveys proposed by the Demographic Health Survey program [14,15].

### Ethics approval and consent to participate

The authors did not need participant consent or ethics approval because they used secondary data from the 2022 KDHS. However, the authors were given access to the 2022 KDHS dataset by registering on the https://www.dhsprogram.com website.

## Results

### Characteristics of the study population

The study analyzed 3,146 children under five years, with the vast majority (79.2%) aged 25–59 months and an almost equal gender split (50.9% male, 49.1% female). Despite widespread ownership of treated mosquito nets (72.6% of households), 10.6% of children did not sleep under a net the previous night. Most mothers were aged 25–34 years (50.8%), and a significant majority of participants lived in rural areas (66%). Regarding education, the largest group of mothers had completed primary school (37.5%). Household wealth varied, with 32.1% classified as poorer and 15.8% as richer. Mothers’ employment was nearly balanced, with 51% not working and 49% working. Most households (85.7%) had two or fewer children under five, and media exposure was high at 76.4%. The majority of children were born in health facilities (88.3%), and just over half (53%) were currently breastfeeding. At the community level, 71% lived in highly educated communities, while 54% resided in high-poverty areas (Table 1).

**Table 1:** Demographic features of the study participants (n = 3,146) in Kenya for the year 2022.

Characteristics	Weighted frequency	Percent
**Age of child in months**		
0-6	125	3.97%
7-12	223	7.09%
13-24	306	9.73%
25-59	2492	79.21%
**Sex of the child**		
Male	1601	50.89%
Female	1545	49.11%
**Slept in Mosquito bed net**		
No	375	10.61%
All children	1774	57.94%
Some children	270	8.82%
No net in the household	693	22.63%
**Maternal age**		
15–24 years	870	27.65%
25–34 years	1598	50.79%
35–49 years	678	21.55%
**Place of residence**		
Urban	1071	34.04%
Rural	2075	65.96%
**Maternal educational status**		
No education	638	20.28%
Primary school	1180	37.51%
Secondary	927	29.47%
Higher	401	12.75%
**Household’s Wealth Status**		
Poorer	1011	32.14%
Poor	550	17.48%
Middle	576	12.87%
Rich	604	19.20%
Richer	405	15.82%
**Types of mosquito bed nets**		
No net	688	21.87%
Only treated net	2285	72.63%
Only the untreated net	173	5.50%
**Mother’s Current working status**		
No	1605	51.01%
Yes	1541	48.98%
**Number of under-five children in the households**		
≤ 2 children	2696	85.70%
> 2children	450	14.30%
**Media exposure**		
Exposed	2404	76.41%
Not exposed	742	23.59%
**Place of delivery**		
Home	367	11.67%
Health facility	2779	88.33%
**Breastfeeding status**		
No	1480	47.04%
Yes	1666	52.96%
**Community-level factors**		
**Community-level educational status**		
Community high level of education	2235	71.04%
Community with a low level of education	911	28.96%
**Community level of poverty**		
Low level	1143	45.55%
High level	1713	54.45%

### Prevalence of Malaria in under-five children

**Fig 1:**
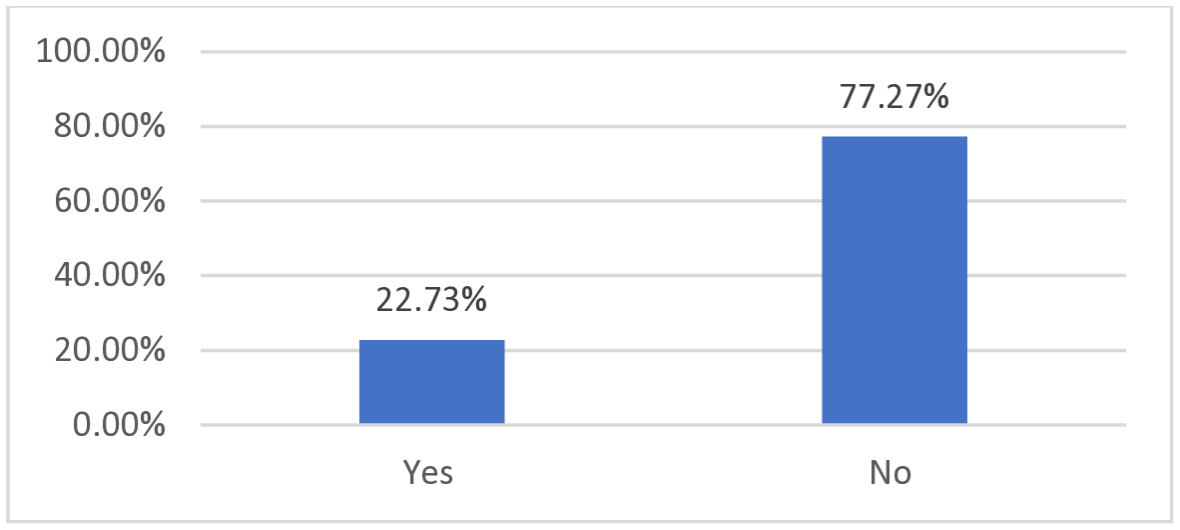
Malaria prevalence among under-five children in Kenya.

The prevalence of malaria among children under five years in Kenya was 22.7% (95% CI: 21.29, 24.22) (Fig 1).

### Model comparison

To assess the extent of variation in the outcome across communities, the study compared three random effects measures: the intraclass correlation coefficient (ICC), median odds ratio (MOR), and proportional change in variance (PCV). The ICC was used to measure the degree of variability between communities. Since individuals within the same community share similar environments, they are more likely to experience similar outcomes compared to those in different communities. The null model revealed an ICC of 30.7%, indicating that approximately 31% of the variation is due to differences between communities.

The study further examined how community-level factors influence the likelihood of the outcome using the median odds ratio (MOR) from the null model, which excludes explanatory variables. The MOR of 1.81 suggests that if two individuals with similar characteristics come from different communities, one high risk and one low risk, the individual from the high-risk community is 81% more likely to experience the outcome. This highlights the significant impact of community-level factors on the likelihood of malaria risk.

Additionally, the proportional change in variance (PCV) was calculated to understand how much of the variation is explained by the inclusion of explanatory variables. The analysis showed that Model I, which incorporates some predictors, explained 5.2% of the variation in the outcome, while Model II and Model III explained 2.4% and 0.6% of the variance, respectively. Model I demonstrated the highest reduction in variance, indicating it captures the most variability.

In terms of model fit, the Log-likelihood ratio (LLR), Deviance Information Criterion (DIC), Akaike Information Criterion (AIC), and Bayesian Information Criterion (BIC) were used to evaluate the models. Model III showed the best fit based on its low AIC and BIC values alongside its significant explanatory power (Table 2).

**Table 2:** Comprehensive Comparison of Models and Outputs of Fitness Parameters for Assessing Malaria in Under-five Children in Kenya.

Fitness parameter	Null model	Model I	Model II	Model III
Community level variance	1.46	1.53	1.82	1.56
ICC	30.7%	29.3%	28.5%	28.2%
MOR	1.81[1.65,2.01]	1.65 [1.40,1.94]	1.79 [1.54, 2.09]	1.66 [1.41, 1.95]
PCV [16]	Reference	5.2%	2.4%	0.6%
**Model fitness parameters**				
Log- likelihood ratio (LLR)	−1629.49	−1351.68	−1404.27	−1347.79
DIC(−2LLR)	3,258.99	2,703.37	2,808.54	2,695.59
AIC	3262.99	2753.37	2816.54	2749.48
BIC	3275.101	2910.72	2912.93	2840.75

**NOTE**: **ICC**, intra-cluster correlation; **MOR**, median odds ratio; DIC, deviance information criterion. The null model is the empty model, the base model without any determining variables. **Model I** is adjusted for individual-level factors. **Model II** is adjusted for community-level factors**. Model III** is the final model adjusted for both individual and community-level factors.

### Factors associated with malaria diseases

Prior to conducting the multivariable multilevel logistic regression analysis, a bivariable (univariate) analysis was performed to assess the crude association between each independent variable and malaria infection. Variables with a p-value less than 0.2 in the bivariable analysis were considered for inclusion in the multivariable model.

The analysis revealed several statistically significant variables. Maternal age showed that mothers aged 25–34 years had reduced odds of the outcome, with an odds ratio of 0.759 (95% CI: 0.578–0.996), indicating a lower risk compared to the reference group of mothers aged 15–24 years. The odds for mothers aged 35–49 years were 0.725 (95% CI: 0.513–1.025), which was not statistically significant. For mosquito bednet type, the use of an untreated net significantly reduced the odds of the outcome, with an odds ratio of 0.398 (95% CI: 0.177–0.892), indicating a lower risk compared to those with no net. However, using a treated net (odds ratio 1.331) did not show statistical significance. The wealth index revealed that the “richest” category had significantly lower odds of the outcome, with an odds ratio of 0.486 (95% CI: 0.263–0.897), suggesting a reduced risk compared to the poorest group. Regarding breastfeeding, children who were breastfed had significantly lower odds of the outcome, with an odds ratio of 0.666 (95% CI: 0.520–0.853). Additionally, households without a mosquito net showed a significantly lower likelihood of the outcome, with an odds ratio of 0.490 (95%CI: 0.310–0.775). At the community level, communities with high poverty had significantly higher odds of the outcome, with an odds ratio of 1.823 (95% CI: 1.196–2.779) (Table 3).

**Table 3:** Multivariable multilevel logistic analysis of malaria diseases among children under five years in Kenya.

Variable Name	Odds Ratio	p-value	95% CI
Intercept	0.432	0.012	0.222-0.842
Individual-level factor			
Maternalage			
15-24	1		
25-34	0.759	0.047	0.578 - 0.996
35-49	0.725	0.069	0.513 - 1.025
Mosquito bednet type			
No net	1		
Only the untreated net	1.331	0.176	0 .879 −2.016
Only treated net	0.398	0.025	0.177 - 0.892
wealth index			
Poorest	1		
Poorer	0.975	0.890	0.688 −1.381
Middle	1.270	0.271	0.82 −1.946
Richer	0.948	0.839	0.569- 1.579
Richest	0.486	0.021	0.263 −0.897
Breast feeding			
Yes	0.666	0.001	0.520 - 0.853
No	1		
The child slept under a mosquito bed net			
No	1		
All children	0.962	0.865	0.619 −1.495
Some children	1.648	0.074	0.951 - 2.854
No net in the household	0.490	0.002	0.310- 0.775
Community-level factor			
Community poverty			
A community with high poverty	1.823	0.005	1.196 −2.779
A community with low poverty			

## Discussion

Malaria remains a significant public health challenge in Kenya, particularly among children under five. The prevalence of malaria in this group was found to be 22.72% (95% CI: 21.29, 24.22), aligning with previous studies that highlight the high burden of malaria among vulnerable populations. This prevalence is higher than studies conducted in Ethiopia, where malaria prevalence among under-five children was reported to be 13.2% [17], and Uganda, which reported a prevalence of 16.8% [18]. However, it is lower than studies conducted in some malaria-endemic areas in Nigeria (27.5%) [19] and the Democratic Republic of the Congo (30.4%) [20]. The observed variations in malaria prevalence across different regions may be attributed to differences in climatic conditions, mosquito breeding sites, malaria control interventions, healthcare access, and socioeconomic status.

This study identified several significant factors associated with malaria prevalence among under-five children. Maternal age was found to be a protective factor, with children born to mothers aged 25–34 years having lower odds of malaria infection compared to younger mothers. This finding aligns with research conducted in Cameroon [21], where older mothers were found to have greater experience and knowledge of malaria prevention measures, such as proper bed net usage and seeking prompt treatment for febrile illnesses.

Household wealth status also played a crucial role in malaria prevalence, with children from the richest households having a lower likelihood of malaria infection. This finding is consistent with studies from Ghana [22] and Burkina Faso [23], which indicated that wealthier families are more likely to afford insecticide-treated bed nets (ITNs), live in better housing conditions, and have improved access to healthcare services, thereby reducing malaria risk.

The use of mosquito bed nets was identified as a significant protective factor. Individuals who sleep under only treated mosquito nets have about 60% lower odds of experiencing malaria compared to those with no net. This provides strong evidence that treated nets are effective in reducing malaria risk. It supports public health efforts to distribute insecticide-treated bed nets (ITNs) rather than untreated ones.This finding aligns with studies conducted in Malawi [24] and Burkina Faso [25], which emphasized the effectiveness of ITNs in reducing malaria transmission. The role of community-level poverty was also significant, with children living in poorer communities being at a higher risk of malaria infection. This association has been supported by studies in sub-Saharan Africa [24], highlighting the influence of economic disparities on malaria burden.

Breastfeeding was found to be another protective factor, as children who were breastfed had lower odds of malaria infection. This result is similar to findings from Nigeria [25], where breastfeeding was associated with better immune responses in infants, potentially reducing their susceptibility to malaria.

### Implications and future research

The findings highlight the need for targeted malaria prevention strategies among under-five children in Kenya, with a focus on maternal and household factors. Strengthening public health interventions, such as improving access to treated mosquito nets, enhancing socioeconomic conditions, and promoting breastfeeding, can significantly reduce malaria prevalence. Policymakers should also consider community-level poverty alleviation programs, as economic disparities increase vulnerability. Health promotion efforts should emphasize educating mothers on malaria prevention and ensuring access to preventive measures, particularly for lower-income families.

### Strengths and limitations of the study

This study has several advantages. First off, the findings’ statistical power and generalizability were probably improved by the utilization of large, nationally representative data. Additionally, a multilevel statistical model was used to correctly address the survey data’s hierarchical nature. But there are restrictions as well. The study’s cross-sectional methodology makes it impossible to establish a causal link between malaria and other factors. Another inherent disadvantage is social desirability bias, which causes individuals to underreport unfavorable actions. The study used information from a secondary survey to address this. Finally, additional potentially significant behavioral and cultural aspects are not taken into account in this study.

## Conclusion

In Kenya, malaria is still a major public health issue for children under five, and bed net use, maternal characteristics, and socioeconomic level all play important roles. According to the study, malaria prevalence can be significantly reduced by enhancing maternal education, economic empowerment, and access to efficient mosquito preventive techniques. Malaria control initiatives should prioritize addressing poverty at the community level and guaranteeing that treated mosquito nets are widely used.

## Abbreviations

AIC: Akaike information criteria
AOR: Adjusted Odds Ratio
BIC: Bayesian information criteria
DIC: Deviance Information Criterion
ICC: Intra-Class Correlation
LLR: Log- Likelihood Ratio
MOR: Median Odds Ratio
PCV: Proportion of Variance Change
CI: confidence interval
AOR: Adjusted odds ratio
EA: enumeration areas
KM: Kilometer
KDHS: Kenya Demographic health survey
